# Widespread Oseltamivir Resistance in Influenza A Viruses (H1N1), South Africa 

**DOI:** 10.3201/eid1411.080958

**Published:** 2008-11

**Authors:** Terry G. Besselaar, Dhamari Naidoo, Amelia Buys, Vicky Gregory, Jo McAnerney, Jack M. Manamela, Lucille Blumberg, Barry D. Schoub

**Affiliations:** National Institute for Communicable Diseases of the National Health Laboratory Service, Sandringham, South Africa (T.G. Besselaar, D. Naidoo, A. Buys, J. McAnerney, J.M. Manamela, L. Blumberg, B.D. Schoub); National Institute for Medical Research, London, UK (V. Gregory)

**Keywords:** Human influenza, oseltamivir, antiviral resistance, South Africa, letter

**To the Editor:** Oseltamivir is the most widely used antiviral drug for influenza; it is a potent inhibitor of influenza virus neuraminidase (NA) protein (*1*). Until recently, oseltamivir resistance occurred in <1% of circulating viruses globally. An increased number of influenza A viruses (H1N1) with resistance to oseltamivir was first reported to the World Health Organization (WHO) by Norway in late January 2008. The viruses carried a specific histidine-to-tyrosine mutation at position 274 (H274Y; H275Y in N1 numbering system) in the NA protein that confers high-level resistance to oseltamivir (*2*). Further surveillance by the European Surveillance Network for Vigilance against Viral Resistance and the WHO Global Influenza Surveillance Network (GISN) showed that 16% of community isolates (0%–67% by country) of influenza A viruses (H1N1) circulating in the 2007–08 season in several other countries were also oseltamivir resistant (*3*). The predominant influenza subtype circulating in South Africa this winter season is H1N1. To determine whether oseltamivir-resistant viruses have spread to South Africa, we examined influenza A (H1N1) isolated during the 2008 winter season for resistance to this antiviral compound.

Specimens were obtained mainly from the National Institute of Communicable Diseases (NICD) active sentinel surveillance program in all 9 provinces. Throat or nasopharyngeal swabs were taken from patients within 48–72 hours of onset of symptoms and sent to NICD laboratories for virus isolation as described (*4*).

Of the H1N1 subtype viruses isolated in May and June, 23 were sent to the WHO Collaborating Centers for Reference and Research on Influenza in London and Melbourne for resistance testing (*5*,*6*). Forty-five of the viruses, which included viruses isolated in July, were tested at NICD by using a modified amplification refractory mutation system PCR (ARMS-PCR) (*7*). This method can simultaneously detect wild-type or mutant virus with the 274 mutation in a single PCR. Partial sequencing of the NA and hemagglutinin (HA) genes was performed to confirm the NA H274Y resistance mutation and to determine genetic drift in HA from the A/Brisbane/59/2007 virus recommended for the Northern Hemisphere 2007–08 vaccine.

At the time of resistance testing, 92 H1N1 subtype viruses had been isolated. The 23 virus isolates sent to the WHO Collaborating Centers were highly resistant to oseltamivir by the NA inhibition enzyme assay, with 50% inhibitory concentration values of 554 nM to 1,485 nM (A. Hay, I. Barr, pers. comm.). All 45 isolates tested locally were positive by ARMS-PCR for oseltamivir resistance at position 274. The H274Y mutation was confirmed by sequence analysis of the N1 genes. The N1 sequences were closely related to those isolated in Europe and elsewhere in the 2007–08 winter season. However, the presence of 1 or 2 aa mutations in viruses from South Africa (M23L and N73K in the stalk region) compared with resistant European isolates indicated that some genetic drift of N1 from the older strains had occurred. Although most 2008 isolates were closely related to the A/Brisbane/59/2007 strain, several of the isolates from South Africa had mutations in an additional 2 or 3 aa residues at positions 183, 185, and 189, which mapped close to the receptor binding site of HA. (GenBank accession numbers for nucleotide sequences obtained in this study are EU914901–EU914916.)

Before the 2007–08 Northern Hemisphere winter, surveillance by GISN laboratories showed that oseltamivir-resistant H1N1 subtype viruses were extremely rare. Low numbers of drug-resistant viruses carrying the H274Y mutation usually followed oseltamivir treatment and showed reduced fitness with poor transmission (*8*). Consequently, fitter nonresistant viruses appear to have predominated. In contrast, no evidence indicated that persons from whom resistant viruses were isolated during the European 2007–08 winter season had either been treated or been in close contact with another person who had been treated with oseltamivir (*2*,*8*).

We report oseltamivir-resistant H1N1 subtype viruses in Africa and the Southern Hemisphere. It appears that resistant viruses have spread from the Northern Hemisphere and have undergone extensive transmission within the population. These viruses may soon appear in other countries in the Southern Hemisphere. Ongoing monitoring is needed to understand the further evolution of oseltamivir resistance.

Clinical symptoms of all patients in this study suggest an illness similar to that generally associated with seasonal influenza A virus (H1N1); no complications were reported. This finding is not unexpected because all isolates tested in the study were from outpatients. The policy in South Africa for use of oseltamivir for treatment of severe influenza remains unchanged because 2008 H3N2 subtype viruses are still drug sensitive.
